# Association of microRNA-224-3p and microRNA-155-5p expressions with plasma long pentraxin 3 concentration and coronary microvascular obstruction following primary angioplasty for acute ST-segment elevation myocardial infarction

**DOI:** 10.1186/s13104-020-05329-2

**Published:** 2020-10-29

**Authors:** Surya Dharma, Iwan Dakota, Shoma Wijaya, Elok Ekawati, Renan Sukmawan, Bambang Budi Siswanto

**Affiliations:** 1 Department of Cardiology and Vascular Medicine, Faculty of Medicine, University of Indonesia, Indonesian Cardiovascular Research Center, National Cardiovascular Center Harapan Kita, Jakarta, Indonesia; 2 Department of Cardiology and Vascular Medicine, Faculty of Medicine, University of Indonesia, National Cardiovascular Center Harapan Kita, Jakarta, Indonesia; 3 Indonesian Cardiovascular Research Center, National Cardiovascular Center Harapan Kita, Jakarta, Indonesia

**Keywords:** Inflammation, microRNA, MVO, STEMI

## Abstract

**Objective:**

Pro-inflammatory stimuli induce a variety set of microRNAs (miRs) expression that regulate long pentraxin-3 (PTX3) protein, which associates with a procoagulant state in the endothelial cells. We evaluated, for the first time in human, the association of miR-224-3p and miR-155-5p expressions with plasma PTX3 concentration and coronary microvascular obstruction (MVO) in patients with acute ST-segment elevation myocardial infarction (STEMI) with symptom onset ≤ 12 h and treated by primary angioplasty. Blood samples for miRs and PTX3 measurement were drawn at emergency department presentation, and were measured by TaqMan real-time PCR and human ELISA kit, respectively.

**Results:**

Of the 217 patients (median age: 54 years, male: 88%), 130 (60%) had angiographic MVO. Spearman analysis showed no correlation between miR-224-3p and miR-155-5p expressions with plasma PTX3 concentration. After adjustment with sex, age, diabetes mellitus, and plasma PTX3 concentration, miR-224-3p ≥ median group was associated with angiographic MVO (odds ratio, 2.60; 95% confidence interval, 1.24 to 5.44, p = 0.01). This study suggests that miR-224-3p and miR-155-5p expressions did not correlate with plasma PTX3 concentration. However, miR-224-3p expression associates with angiographic MVO following primary angioplasty for STEMI. Future studies are needed to identify the specific gene/protein related with miR-224-3p expression in MVO.

## Introduction

Coronary microvascular obstruction (MVO) during primary percutaneous coronary intervention (PCI) for ST-segment elevation myocardial infarction (STEMI) associates with a worse clinical outcome [1]. Interestingly, the genetic profile and PTX3 concentration have been known to be associated with coronary MVO [2, 3]. Understanding the basic molecular mechanism of MVO is essential for possible therapeutic target, e.g., identifying the specific microRNA (miR) that associates with coronary MVO. Animal studies showed that proinflammatory stimuli induces several miRs expresion that have been identified to regulate long pentraxin-3 (PTX3) gene expression including miR-224, miR-146, miR-155 [4] and miR-150 [5]. Of these, miR-224 and miR-155 expression were acutely increased > twofold following an inflammation stimuli [4]. It is known that PTX3 protein expression causing a procoagulant environment in the endothelial cells [6], thus higher plasma PTX3 concentration has been known to be associated with thrombus grade 4 or 5 in patients with acute STEMI [3]. However, whether miR-224-3p and miR-155-5p associate with PTX3 concentration and coronary MVO following primary angioplasty for patients with acute STEMI are unknown. Considering miR-155-5p and miR-224-3p are strongly involve in inflammation and regulate PTX3 expression, we evaluated for the first time in human, the association of miR-155-5p and miR-224-3p expressions with plasma PTX3 concentration and angiographic MVO in patients with acute STEMI treated by primary angioplasty.

## Main text

### Material and methods

We performed a cross sectional study in 335 consecutive patients with acute STEMI with symptom onset ≤ 12 h treated with primary angioplasty in our hospital between January 1, 2018 and August 2, 2018. Patients with no adequate coronary angiogram images for myocardial blush grade (MBG) evaluation after primary angioplasty were excluded, and final analysis were performed in 217 patients. Blood samples for PTX3 measurement and miRs expression were drawn at emergency department presentation, before primary PCI.

### Primary PCI procedure

Primary PCI procedure was previously described [3]. In brief, all patients were pre-treated with 160–320 mg acetylsalicylic acid and 600 mg clopidogrel or 180 mg ticagrelor orally. Unfractionated heparin (100 IU/kg) was administered intravenously in the catheterization laboratory after sheath insertion. During primary PCI, an initial coronary angiogram was performed to assess the infarct-related artery (IRA), baseline thrombolysis in myocardial infarction (TIMI) flow, and thrombus grade. After primary PCI, the final TIMI flow and MBG were evaluated using standard technique.

### microRNA measurement

Blood samples were collected in EDTA vacuum containers, followed by blood centrifugation for 15 min at 2500 rpm within 30 min of blood collection. The peripheral blood mononuclear cell (leukocyte cell) was isolated by centrifugation at 2500 rpm within 30 min of blood collection at 4 °C in Lymphoprep™ (Stem Cell Technology, Oslo, Norway). The RNA was then isolated using a miReasy Mini Isolation Kit (Qiagen, Hilden, Germany) according to the manufacturer's protocol. The total RNA was isolated from 200 µl cells (2 × 10^6^), and determined by Multiskan Sky Reader (Thermo Fisher Scientific, Massachusetts, US). The 2 ng of total RNA was used for Reverse Transcription PCR (Applied Biosystem, Carlsbad, CA). To allow for normalization of sample-to-sample variation in RNA isolation and reverse transcription, RNU44 was used as an endogenous control. The expression of miR-224-3p, miR-155-5p, and RNU44 were quantified using TaqMan miR real-time PCR kit (Applied Biosystem, Carlsbad, CA) following the fluoresce (FAM) signal in 7500 Fast Real-Time PCR (Applied Biosystem, Carlsbad, CA).

All miR measurements were performed in duplicate by investigators who were unaware of the patient’s characteristics and outcome. microRNA-224-3p and miR-155-5p expressions are given as Cycle threshold (Ct). To determine ∆Ct, the Ct of miR-224-3p and miR-155-5p were normalized with RNU44. The ∆Ct miR-224-3p and miR-155-5p were reduced by ∆Ct of healthy volunteers to determine the ΔΔCt value. The relative expression was calculated by 2^–ΔΔCt^.

### Plasma PTX3 measurement

As previously described [3], PTX3 was assayed with a commercial Human PTX3 ELISA kit (San Diego, California, USA) by dedicated molecular biology laboratory personnel and blinded to the characteristics of the patients.

### Study outcome

The primary outcome of the study was the correlation between miR-224-3p and miR-155-5p expressions with plasma PTX3 concentration. Secondary outcome was the occurrence of angiographic MVO, defined as a post-primary PCI TIMI grade < 3 flow or TIMI grade 3 flow with MBG 0 or 1.

### Definition

The diagnosis of STEMI was made based on the presence of ischemic symptoms (> 20 min) and persistent ST-segment elevation in at least two contiguous leads, a new left bundle-branch block, or a true posterior myocardial infarction confirmed by posterior leads [7].

Thrombus grade was determined using the TIMI scoring system and divided into 0,1,2,3,4 or 5 [8], TIMI flow grade was classified into 0,1,2 or 3 [9], and MBG was classified into 0,1,2 or 3 [10].

All patients gave written informed consent, and the study approved by the institutional review board of the National Cardiovascular Center Harapan Kita, Jakarta, Indonesia.

### Statistical analysis

The miRs expressions were analyzed based on the median values. Patients were grouped and compared by the median values of miRs expression (miR-155-5p ≥ median group vs. miR-155-5p < median group, and miR-224-3p ≥ median group vs. miR-224-3p < median group). Continuous data are presented as median (interquartile range) and compared by Mann–Whitney U-test. Categorical data are presented as percentages and differences were compared by Chi-square test or Fischer exact test as appropriate.

Spearman correlation tests were used to find the correlation between miR-224-3p and miR-155-5p expressions with plasma PTX3 concentration. Logistic regression analyses were performed using Back-wald method to find the association of miRs expressions, and selected variables with angiographic MVO. Variables included into the multivariate analysis were age, sex, diabetes mellitus, miR-155-5p and miR-224-3p expressions, and plasma PTX3 concentration. The cut-off of PTX3 concentration (≥ 0.26 ng/ml) for the optimal prediction of coronary MVO was calculated using the receiver operating characteristics (ROC) curve [a sensitivity of 68%, a specificity of 68%, and an area under the curve (AUC) of 0.69 (95% confidence interval: 0.62–0.76)] (Additional file 1: Figure S2). No statistically significant differences in ROC-AUC were found for the miR-224 and miR-155.

A p-value of < 0.05 was considered statistically significant. All statistical analyses were performed using a statistical package (SPSS version 17, SPSS Inc. Chicago, IL, USA).

## Results

### Patient characteristics

Of 217 Indonesian patients, the median age was 54 years, 191 (88%) were male, and 130 (60%) had angiographic MVO. The median fold-increase of miR-224-3p and miR-155-5p were 0.79 (0.26 – 3.74) and 1.35 (0.38–4.10), respectively. In general, the characteristics of patients were similar between the groups. Left-circumflex IRA was more often in the miR-224-3p ≥ median group compared with miR-224-3p < median group (6.4% vs. 0%, p = 0.01). Patient’s characteristic is displayed in Table 1.

**Table 1:** Patient characteristics

Variables	All patients (N = 217)	miR-224-3p ≥ median (N = 109)	miR-224-3p < median (N = 108)	*P*	miR-155-5p ≥ median (N = 109)	miR-155-5p < median (N = 108)	*P*
Clinical characteristics							
Age (y)	54 (47–63)	55 (48–63)	53 (46–63)	0.38	56 (48–67)	52 (46–63)	0.22
Male, n (%)	191 (88)	99 (90.8)	92 (85.2)	0.20	93 (85.3)	98 (90.7)	0.21
Body mass index, kg/m^2^	24.2 (22.2–26.9)	23.8 (21.5–26.8)	24.8 (22.5–27.1)	0.33	24.2(21.4–27.1)	23.8 (22.4–26.6)	0.62
CAD risk factors, n (%)							
Hypertension	129 (59.4)	68 (62.4)	61 (56.5)	0.37	66 (60.6)	63 (58.3)	0.73
Diabetes mellitus	65 (30)	31 (28.4)	34 (31.5)	0.62	30 (27.5)	35 (32.4)	0.43
Dyslipidemia	53 (24.4)	29 (26.6)	24 (22.2)	0.45	28 (25.7)	25 (23.1)	0.66
Smoking	168 (77.4)	86 (78.9)	82 (75.9)	0.60	81 (74.3)	87 (80.6)	0.27
Family History	34 (15.7)	14 (12.8)	20 (18.5)	0.25	19 (17.6)	15 (13.8)	0.43
Symptom onset > 6 h, n (%)	105 (48.4)	48 (44.0)	57 (52.8)	0.19	48 (44.0)	57 (52.8)	0.19
Anterior MI, n (%)	112 (51.6)	56 (51.4)	56 (51.9)	0.94	58 (53.2)	54 (50.0)	0.63
TIMI risk score > 4, n (%)	61 (28.1)	31 (28.4)	30 (27.8)	0.91	33 (30.3)	28 (25.9)	0.47
Killip class II–IV, n (%)	41 (18.9)	17 (15.6)	24 (22.2)	0.21	17 (15.6)	24 (22.2)	0.21
Medication at 24 h, n (%)							
Salicylic acid	217 (100)	109 (100)	108 (100)	NA	109 (100)	108 (100)	NA
Clopidogrel	164 (75.6)	83 (76.1)	81 (75.0)	0.84	81 (74.3)	83 (76.9)	0.66
Ticagrelor	53 (24.4)	26 (23.9)	27 (25)	0.84	28 (25.7)	25 (23.1)	0.66
ACE-inhibitor	60 (27.6)	24 (22.0)	36 (33.3)	0.06	28 (25.7)	32 (29.6)	0.51
Beta-blocker	4 (1.8)	2 (1.8)	1 (1.9)	1.00	1 (0.9)	3 (2.8)	0.30
Statin	212 (97.7)	106 (97.2)	106 (98.1)	1.00	107 (98.2)	105 (97.2)	0.68
Anticoagulation, n (%)	66 (30.4)	35 (32.1)	31 (28.7)	0.58	33 (30.2)	33 (30.5)	0.96
Medications at discharged, n (%)							
Salicylic acid	214 (28.6)	108 (99.1)	106 (98.1)	0.62	108 (99.1)	106 (98.1)	0.62
Clopidogrel	164 (75.6)	86 (78.9)	78 (72.2)	0.25	82 (75.9)	82 (75.9)	0.90
Ticagrelor	52 (24.1)	23 (21.6)	29 (26.9)	0.34	27 (24.8)	25 (23.4)	0.80
ACE-inhibitor	189 (87.1)	95 (87.2)	94 (87.0)	0.97	95 (87.2)	94 (87.0)	0.97
Beta-blocker	188 (86.2)	95 (87.2)	93 (86.1)	0.81	96 (88.1)	92 (85.2)	0.53
Statin	217 (100)	109 (100)	108 (100)	NA	109 (100)	108 (100)	NA
Echocardiography data							
LVEF, (%)	48 (40–56)	48 (41–56)	48 (40–56)	0.36	50 (41–57.7)	46 (39–55)	0.02
Laboratory characteristics							
Hemoglobin, g/dl	14.4 (13–15.4)	14.6 (13.3–15.5)	13.9 (12.8–15.2)	0.48	14.3 (13.1–15.4)	14.6 (13–15.3)	0.30
Leukocyte count, /ul	13,230 (11,050–16,130)	14,090 (11,140–15,990)	12,905 (10,962.5–16,265)	0.68	13,570 (10,992.5–16,255)	13,230 (11,250–16,130)	0.64
Baseline creatinine > 1.3 mg/dl, n (%)	38 (16.6)	13 (11.9)	23 (21.3)	0.06	18 (16.5)	18 (16.7)	0.97
Admission blood glucose level, mg/dl	141 (117–201)	138 (115–195)	144.5 (118.2–207.2)	0.44	135 (115–199.7)	144 (119–202)	0.11
Initial Troponin T, ng/L	398 (155–1021)	418 (168–1714)	389 (139.2–691)	0.16	319 (152.2–1256)	417 (155–962)	0.79
Primary PCI characteristics							
Baseline TIMI flow, n (%)							
0	160 (73.7)	77 (70.6)	83 (76.9)	0.29	77 (70.6)	83 (76.9)	0.29
1	11 (5.1)	5 (4.6)	6 (5.6)	0.74	6 (5.5)	5 (4.6)	0.76
2	30 (13.8)	19 (17.4)	11 (10.2)	0.12	17 (15.6)	13 (12.0)	0.44
3	16 (7.4)	8 (7.3)	8 (7.4)	0.98	9 (8.3)	7 (6.5)	0.61
Final TIMI flow, n (%)							
0	2 (0.9)	0	2 (1.9)	0.34	1 (0.9)	1 (0.9)	1.00
1	16 (7.4)	8 (7.3)	8 (7.4)	0.98	10 (9.2)	6 (5.6)	0.30
2	98 (45.2)	57 (52.3)	41 (38.0)	0.03	48 (44.0)	50 (46.3)	0.73
3	101 (46.5)	44 (40.4)	57 (52.8)	0.06	50 (45.9)	51 (47.2)	0.82
Thrombus grade 4&5, n (%)	169 (77.9)	83 (76.1)	86 (79.6)	0.53	84 (77.1)	85 (78.7)	0.77
Door-to-device time, min	64 (54–79)	67 (56–80)	63 (53–73	0.07	68 (56.5–83.7)	62 (52–73)	0.01
Procedural time, min	40 (31–55)	40 (31–57)	40 (30–52)	0.39	40 (31.2- 56.5)	41 (29–52)	0.59
Radial access, n (%)	184 (84.8)	90 (82.6)	94 (87.0)	0.35	89 (81.7)	95 (88)	0.19
Balloon predilation, n (%)	208 (95.9)	105(96.3)	103 (95.4)	0.74	103 (94.5)	105 (97.2)	0.49
Manual thrombectomy, n (%)	3 (2.8)	3 (2.8)	0	0.24	2 (1.8)	1 (0.9)	1.00
Intracoronary eptifibatide, n (%)	10 (4.6)	5 (4.6)	5 (4.6)	1.00	4 (3.7)	6 (5.6)	0.53
Infarct related artery, n (%)							
Left main	0	0	0	0	0	0	0
LAD	113 (52.1)	56 (51.4)	57 (52.8)	0.83	57 (52.3)	56 (51.9)	0.94
LCX	7 (3.2)	7 (6.4)	0 (0)	0.01	1 (0.9)	6 (5.5)	0.05
RCA	97 (44.7)	46 (42.2)	51 (47.2)	0.45	46 (42.2)	51 (47.2)	0.45
Coronary involvement, n (%)							
1-Vessel disease	68 (31.1)	32 (29.4)	36 (33.3)	0.52	35 (32.1)	33 (30.6)	0.80
2-Vessel disease	66 (30.4)	35 (32.1)	31 (28.7)	0.58	33 (30.6)	33 (30.3)	0.96
3-Vessel disease	73 (33.6)	40 (36.7)	33 (30.6)	0.33	38 (34.9)	35 (32.4)	0.70
Left Main disease	10 (4.6)	2 (1.8)	8 (7.4)	0.05	3 (2.8)	7 (6.5)	0.74
Use of mechanical ventilation, n (%)	9 (4.1)	4 (3.7)	5 (4.6)	0.74	4 (3.7)	5 (4.6)	0.74
Use of DES, n (%)	213 (98.2)	107 (98.2)	106 (98.1)	1.00	107 (98.2)	106 (9.1)	1.00

CAD, coronary artery disease; MI, myocardial infarction; TIMI, thrombolysis in myocardial infarction; ACE, angiotensin converting enzyme; LVEF, left ventricular ejection fraction; PCI, percutaneous coronary intervention; LAD, left anterior descending coronary artery; LCX, left circumflex artery; RCA, right coronary artery; DES, drug-eluting stent; NA, not analyzed

### Study outcome

**Fig. 1:**
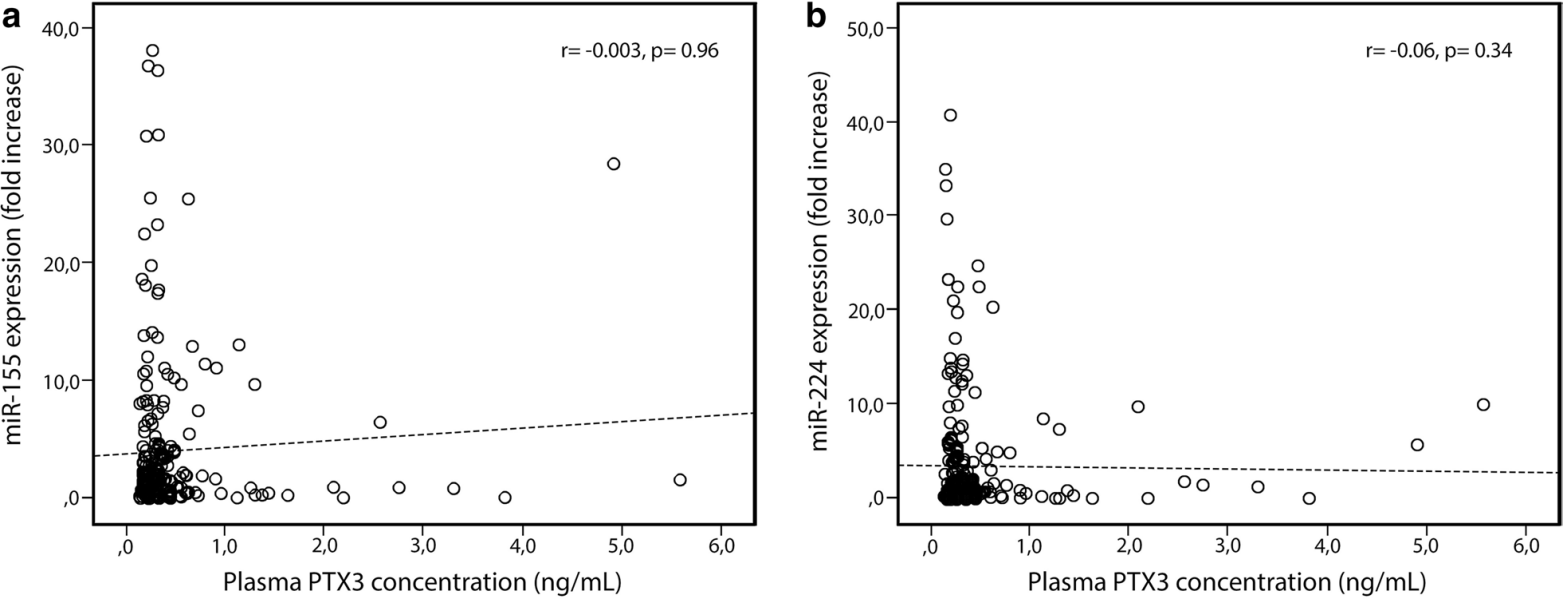
Correlation between miR-155-5p expression (A) and miR-224-3p (B) with plasma PTX3 concentration. PTX3 indicates long pentraxin 3

Correlation analyses showed no correlation between miR-155-5p and miR-224-3p expression with plasma PTX3 concentration (Fig. 1).

The incidence of coronary MVO was higher in patients with miR-224-3p ≥ median group compared with miR-224-3p < median group (67% vs. 52.8%, p = 0.03), and was similar across the miR-155-5p group (59.6% vs. 60.2%) (Additional file 2: Figure S1). Multivariate analysis showed that miR-224-3p ≥ median group was associated with angiographic MVO (adjusted odds ratio (OR), 2.60; 95% confidence interval (CI), 1.24 to 5.44) (Table 2).

**Table 2:** Logistic regression analysis to find association of selected variables with angiographic MVO

	Odds ratio	95% confidence interval	P-value
miR-224-3p ≥ median	2.60	1.24–5.44	0.01
miR-155-5p ≥ median	0.53	0.25–1.10	0.09
Age > 65 years	1.38	0.58–3.28	0.46
Female	1.99	0.73–5.39	0.17
Diabetes mellitus	0.96	0.49–1.87	0.91
PTX3 concentration ≥ 0.26 ng/ml	4.69	2.54–8.65	< 0.001

miR, microRNA; PTX3, long pentraxin 3

## Discussion

Our study demonstrates for the first time in human that miR-224-3p expression (not miR-155-5p) was associated with angiographic MVO in patients with STEMI treated by primary angioplasty. However, the expression level of miR-224-3p and miR-155-5p did not correlate with plasma PTX3 concentration. These findings add several important insights to the knowledge of molecular mechanism of MVO in STEMI.

### Association of microRNA with inflammation and innate-immunity

Advances in epigenetic has led to the identification of miRs expressions in human mononuclear cells that have been used as biomarkers of cardiovascular disease and injury [11, 12]. At present, there are 4312 miRs and 23,426 target genes that have been identified in humans [13], of which ~ 60 miRs are involved in cardiovascular disease [14].

MicroRNAs are endogenous small non coding RNA molecules that consist of approximately 19–25 oligonucleotides, and pair with the 3′ untranslated region (UTR) sites in mRNAs of protein-coding genes to down-regulate gene expression [15]. MicroRNAs play important role in biological mechanisms including cell cycle, cell proliferation, cell differentiation, metabolism, apoptosis and cellular signaling [2].

In our STEMI population, the mechanism by which miR-224-3p expression associates with coronary MVO is unclear, perhaps miR-224-3p pairs with the UTR sites in mRNA of a protein-coding specific gene, resulting in down-regulation of a specific gene expression and/or protein, other than PTX3 gene, that involved in innate immunity during acute inflammation in STEMI. The down regulation of the specific protein probably leads to an excessive inflammatory response during an infarction, thus enhances a procoagulant state in the endothelial cell that possibly related with coronary MVO. Furthermore, among the miR-224 validated targets, some are possibly involved in mechanisms leading to microvascular damage. It is hypothesized that these mechanisms are orchestrated by nuclear factor κB (NF-κB) that activates the complement immune cascade regulates by a specific protein, and suggests that miR-224-3p and the specific protein are involved in a regulatory loop in the NF-κB pathway. It is known that in hepatocellular carcinoma, the miR-224-3p promoter has a binding site for the NF-κB, a key transcription factor in the immune response, which activates miR-224-3p transcription [16].

In this study, we found no correlation between miR-224-3p and miR-155-5p expressions and PTX3 concentration. This is probably related with the genetic variants such as the polymorphism of rs2306519. It is known that polymorphism of rs2306519 is associated with PTX3 concentration [2, 17]. Probably miR-224 and rs2306519 have similar gene target, that is the PTX3 gene. miR-224-3p pairs with the 3′ UTR sites in mRNAs of protein-coding genes to down-regulate PTX3 gene expression, and possibly the 3′ UTR is disrupted by the rs2306519 that may inhibit PTX3 gene expression.

Other possible explanation that PTX3 is derived from other cells rather than the circulating leukocytes where the miR-224 were analyzed. Further studies are needed to explain the exact mechanism.

The multivariate analysis from this study showed that both miR-224-3p expression and plasma PTX3 concentration were each associated with angiographic MVO (OR, 2.60, 95% CI, 1.24 to 5.44, and OR, 4.69, 95% CI, 2.54 to 8.65, respectively) (Table 2). Furthermore, a recent study showed that polymorphism of a certain gene associates with coronary MVO in STEMI [2]. Together, these findings explained the complexity of the molecular mechanism related to coronary MVO in STEMI. Choice of antiplatelet also has been found to be associated with microvascular dysfunction [18]. In our study, the choice of antiplatelet and use of anticoagulant were similar across the studied group (Table 1), and possibly not affecting the results.

### Clinical implication

The results of this study suggest the potential use of miR-224-3p for prognostic marker of MVO in STEMI. We hypothesized that inhibiting the miR-224-3p expression may result in increasing the protein-coding gene expression that involved in innate immunity response during acute inflammation in STEMI, preventing the excessive inflammation and coagulation environment in the endothelium, thus preventing coronary MVO. Further studies are warranted to test the hypothesis. Considering the miR measurement is time consuming and requires specific skill, and limiting its use in daily clinical practice, it is suggested to develop a more quick and easy to use kit that can be used as a bedside test to widening the use of miRs as novel prognostic biomarkers in STEMI.

In conclusion, this study suggests that miR-224-3p and miR-155-5p expressions did not correlate with plasma PTX3 concentration. However, miR-224-3p expression associates with angiographic MVO following primary angioplasty for STEMI. Future studies are needed to identify the specific gene/protein related with miR-224-3p expression in MVO.

## Limitations

There are several limitations of this study. First, this study could not explain the exact molecular mechanism related with miR-224-3p expression and coronary MVO. The proposed molecular mechanisms described were based on several hypotheses that need to be tested in future trials. Second, the MVO criteria used in this study was based on angiographic criteria that corresponding with the cardiac magnetic resonance criteria for MVO. Third, the hematologic parameters that associate with no reflow were not evaluated. Finally, there is lack of an independent validation cohort, and C-statistic analyses failed to show a significant discriminative power of miR-224 and miR-155 for MVO.

## Supplementary information

**Additional file 1: Figure S2.**Receiver operating curve characteristics of PTX3 for predicting coronary MVO. PTX3 indicates long pentraxin-3; MVO, microvascular obstruction.

**Additional file 2: Figure S1.** Incidence of MVO in miR-224-3p and miR-155-5p groups. MVO indicates microvascular obstruction; NS, not significant.

## Data Availability

Data and material are available by contacting the corresponding author on reasonable request. It is necessary to obtain permission from the institutional review board at the National Cardiovascular Center Harapan Kita Hospital to access the data.

## Abbreviations

MVO: Microvascular obstruction
PCI: Percutaneous coronary intervention
STEMI: ST-segment elevation myocardial infarction
miR: MicroRNA
PTX3: Long pentraxin 3
MBG: Myocardial blush grade
